# Exchanging Murine and Human Immunoglobulin Constant Chains Affects the Kinetics and Thermodynamics of Antigen Binding and Chimeric Antibody Autoreactivity

**DOI:** 10.1371/journal.pone.0001310

**Published:** 2007-12-12

**Authors:** Marcela Torres, Narcis Fernandez-Fuentes, András Fiser, Arturo Casadevall

**Affiliations:** 1 Department of Microbiology and Immunology, Albert Einstein College of Medicine, Bronx, New York, United States of America; 2 Department of Biochemistry, Albert Einstein College of Medicine, Bronx, New York, United States of America; Vanderbilt University School of Medicine, USA

## Abstract

Mouse-human chimeric antibodies composed of murine variable (V) and human (C) chains are useful therapeutic reagents. Consequently, we investigated whether heterologous C-regions from mice and humans affected specificity and affinity, and determined the contribution of C_H_ glycosylation to antigen binding. The interaction of a 12-mer peptide mimetic with monoclonal antibody (mAb) 18B7 to *Cryptococcus neoformans* glucuronoxylomannan, and its chimeric (ch) and deglycosylated forms were studied by surface plasmon resonance. The equilibrium and rate association constants for the chAb were higher than for mAb 18B7. V region affinity was not affected by C_H_ region glycosylation whereas heterologous C region of the same isotype altered the Ab binding affinity and the specificity for self-antigens. Structural models displayed local differences that implied changes on the connectivity of residues. These findings suggest that V region conformational changes can be dictated by the C_H_ domains through an allosteric effect involving networks of highly connected amino acids.

## Introduction

Monoclonal antibodies have found a wide range of applications *in vitro* for immunochemical characterization and quantization of antigens (Ags) as well as many different therapeutic applications, for the treatment of microbial, autoimmune and malignant diseases. Although several murine mAb are in clinical use, their use is limited because treated patients develop human anti-murine antibodies (HAMA) that may reduce the effectiveness of a treatment due to the formation of immune complexes. Ideally, human Abs would be used in therapy but these remain difficult to produce. One alternative is the construction and production of mouse-human chAbs. These molecules have human C regions to provide effector functions and mouse V regions that bind antigen. Since the C region constitutes most of the mass of the immunoglobulin (Ig), chAbs are largely human in composition and significantly less immunogenic. However, a central assumption in the construction and use of mouse-human chAbs is that they retain the affinity and specificity of the parental murine mAb. Although this assumption is supported by an overwhelming amount of data showing that V regions interact with Ag, there is now considerable data that the C region can affect V region structure therefore affecting Ab affinity and specificity [1]–[6]. In this regard, the different C_H_ domains can impose diverse structural constrains to the interaction of Ab with Ags, especially multivalent Ags such as polysaccharide. This raises the question of whether similar effects can follow the construction of mouse-human chimeric antibodies where heterologous C regions manifesting differences in sequence are exchanged to create a less immunogenic molecule.

The murine mAb 18B7 is being developed as an adjunctive passive immunotherapy treatment of cryptococcal meningitis in patients with AIDS [7]. Infusion of mAb 18B7 into patients induced HAMA responses, despite their immunosuppressed status [7]. Since cryptococcosis is a chronic disease, there is interest in generating therapeutic Ab reagents that are non-immunogenic and suitable for multiple infusions. Given that the murine mAb 18B7 has undergone clinical testing and that has been extensively studied in the laboratory, one approach was to generate chAbs as potential therapeutic reagents, with the assumption that expression of mAb 18B7 V regions in combination with human C regions would maintain the specificity and affinity of the murine mAb. That assumption was challenged by the observation that a set of mouse-human chAbs derived from mAb 18B7 [8] to the capsular polysaccharide of *C. neoformans* glucuronoxylomannan (GXM), manifested subtle differences in their binding characteristics [1]. Furthermore, there is extensive anecdotal and unpublished evidence that many attempts to generate humanized mAbs based on mouse V regions have failed to produce useful Abs because of loss of specificity or affinity. However, the available data are only suggestive since one can always blame differences in avidity resulting from different hinge region geometry for differences in the binding of chAbs relative to the parental murine mAb.

In this study, we used surface plasmon resonance (SPR) to investigate the thermodynamic and kinetic properties of binding of the GXM-binding mAb 18B7, its deglycosylated form (18B7dg) and its mouse-human chAb counterpart. These Abs have identical V regions but differ in their C domains. Most of the Ig glycosylation sites are found in the C_H_ domains but glycosylation seems to have little if any effect on Ab binding to Ag [9], [10]. Comparison of the binding kinetics and thermodynamics between the glycosylated and deglycosylated murine mAb 18B7 forms and the chAb 18B7 revealed differences in binding affinity attributed to the heterologous C_H_ region, however no contribution from the carbohydrate motif was observed. The results have important implications for the design and use of heterologous V and C chains in therapeutic Abs.

## Results

### Effect of glycosylation and heterologous constant region on the kinetic and equilibrium binding constants

**Figure 1:**
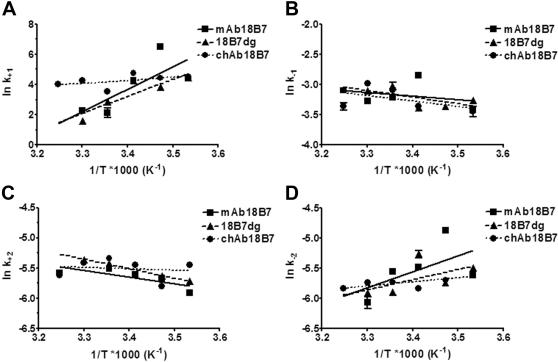
Analysis of the association and dissociation rates constants by SPR. Van't Hoff plots of the rate constants *A*, k_+1_, *B*, k_−1_, *C*, k_+2_ and *D*, k_−2_ for mAb 18B7, 18B7dg, and chAb 18B7 binding to peptide P1. All data points were obtained from the BIAevaluation 4.1 software using a two-state model. Lines represent the best fit to a linear regression using a 95% confidence interval. Errors bars were calculated using propagation of error for SE of each assay.

**Figure 2:**
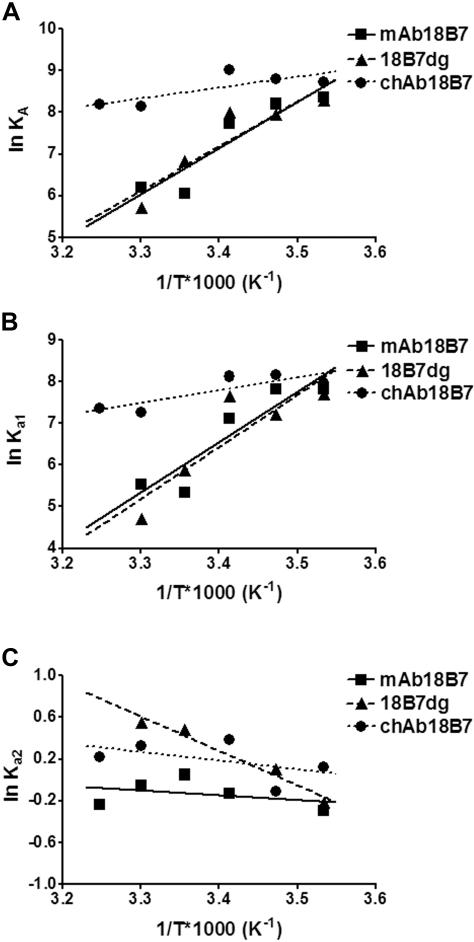
Equilibrium affinity constants for the binding of mAb 18B7, 18B7dg, and chAb 18B7 to peptide P1. Van't Hoff plots of the *A*, equilibrium association constant (K_A_), *B*, encounter equilibrium association constant (K_a1_) and *C*, docking equilibrium association constant (K_a2_) for mAbs 18B7 binding to peptide P1. All equilibrium constants were calculated from the rate constant obtained from the fittings to a two-state model. Lines represent the best fit to a linear regression using a 95% confidence interval. Errors bars were calculated using propagation of error for SE of each assay.

To study the role of glycosylation we compared mAb 18B7, chAb 18B7, and 18B7dg binding to the GXM mimetic peptide P1. Analysis of these mAbs by SPR revealed that the association rate constants for the encounter step (k_+1_) for mAb 18B7 and 18B7dg were similar, but different from chAb 18B7 (**Figure 1A**). The equilibrium dissociation constants (K_D_) for mAb 18B7, 18B7dg and chAb 18B7 are 2.33×10^−3^, 1.07×10^−3^ and 5.41×10^−4^ M at 25°C, respectively. Van't Hoff plots of the interaction of these Abs with peptide P1 revealed that their affinity decreased with temperature (**Figure 2A**). In addition, the equilibrium association constants for the binding of mAb 18B7 and its deglycosylated form to P1 manifested similar affinity constants and their binding characteristics were more influenced by the change in temperature than that observed for chAb 18B7 (**Figure 2A**). It is also noticeable that chAb 18B7 had a higher overall equilibrium affinity constant (K_A_) than the parental mAb 18B7. In addition, for all these complexes, the encounter equilibrium association constant K_a1_ showed a pattern that is almost identical to the equilibrium association constant (**Figure 2B**), while the docking equilibrium association constant K_a2_ was relatively unaffected by changes in temperature, showing similar trends between Ab-peptide complexes (**Figure 2C**).

### Effect of glycosylation and heterologous constant region on the thermodynamic parameters of Ab binding

**Figure 3:**
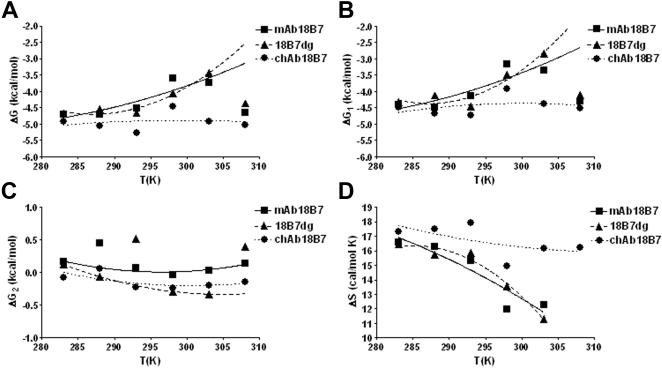
Thermodynamics of binding of mAbs 18B7, 18B7dg and chAb 18B7 to a peptide mimetic of GXM. Behavior of *A*, free energy of binding (ΔG), *B*, encounter free energy of binding (ΔG_1_), *C*, docking free energy of binding (ΔG_2_) and *D*, entropy of binding (ΔS) as a function of temperature for the mAb 18B7, 18B7dg and chAb 18B7 binding to peptide P1. Free energies were calculated from the formula ΔG = −RT ln K_A_, ΔG = −RT ln K_a1_, ΔG = −RT ln K_a2_ and ΔS from the formula ΔG = ΔH−TΔS.

**Figure 4:**
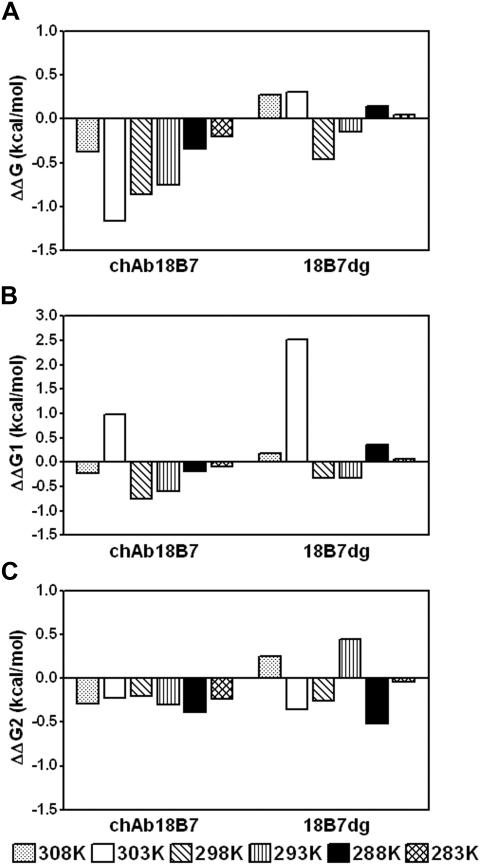
Effect of temperature on the differences in free energy of binding (ΔΔG) of chAb 18B7 and 18B7dg complexes. Each panel corresponds to the ΔG of chAb 18B7-P1 and 18B7dg-P1 complex formation, relative to the ΔG of parental mAb 18B7, at different temperatures. *A*, overall difference of the free energy of binding (ΔΔG) and overall difference of the free energy of binding of *B*, encounter and *C*, docking steps (ΔΔG1 and ΔΔG2) for the mAbs 18B7dg and chAb 18B7 at 35°C, 30°C, 25°C, 20°C, 15°C, 10°C. Values of ΔΔG were calculated as ΔG_ch/dgAb_−ΔG_mAb18B7_.

**Figure 5:**
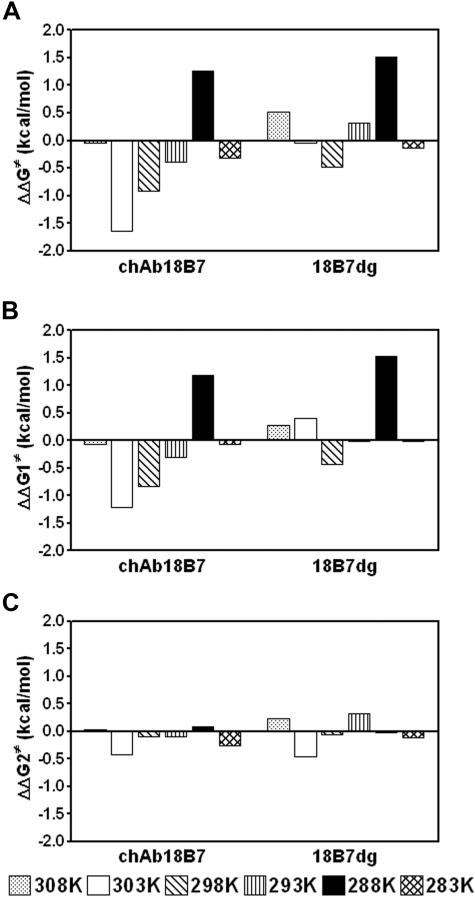
Differences in free activation energy of binding (ΔΔG^‡^) of the transition states of chAb 18B7 and 18B7dg for the binding to peptide P1. Each panel corresponds to the ΔG of chAb 18B7-P1 and 18B7dg-P1 complex formation, relative to the ΔG of parental mAb 18B7. *A*, free activation energy of binding (ΔΔG^‡^), *B*, encounter activation free energies (ΔΔG1^‡^) and *C*, docking activation free energies (ΔΔG2^‡^), as a function of temperature. Values of ΔΔG were calculated as ΔG_ch/dgAb_−ΔG_mAb18B7_. In all cases, the activation energies for the encounter and docking steps (ΔG1^‡^ and ΔG2^‡^) were calculated from k_+1_ and k_+2_, respectively, according to the transition state theory: ΔG^‡^ = −RT ln K^‡^, ΔΔG^‡^ = −RT ln K^‡^_ch/dg Ab_/K^‡^_mAb18B7_, and K^‡^ = *k_B_* T k_a_/*ћ*, where k_a_ is the forward rate constant, *k_B_* is Boltzmanns constant and *ћ* is Plank's constant.

Analysis of the Gibbs free energy of binding (ΔG) profiles of these mAb-peptide complexes showed that for mAb 18B7 and its deglycosylated form, the energy of binding increases with temperature, followed by a decrease at temperatures above 30°C (303 K), whereas the energy of binding for chAb 18B7-P1 complex was relatively insensitive to temperature changes (**Figure 3A**). A similar pattern was observed for the encounter energy of binding (ΔG1) (**Figure 3B**) for the complexes formed by mAb 18B7 and 18B7dg and chAb 18B7 relative to ΔG. The docking energy of binding (ΔG2) increased slightly at low temperatures, but remained otherwise relatively insensitive to changes in temperature, and manifested similar patterns for all Ab-peptide complexes (**Figure 3C**). From these data, we can establish that most of the free energy changes come from the encounter step. Furthermore, ΔS for mAb 18B7 and its deglycosylated form decreased as the temperature increased (**Figure 3D**), but showed a sharp increase above 30°C (303 K). In contrast, ΔS for the chAb 18B7-P1 complex decreased only slightly with increasing temperature (**Figure 3D**). Also, differences in ΔG (ΔΔG) of mAbs 18B7dg and chAb 18B7 relative to mAb 18B7 indicate the interaction formed by chAb 18B7 is energetically more favorable than for mAb 18B7dg (**Figure 4A**), with contributions to the differences in energy of binding coming from the encounter and docking steps (**Figure 4B and 4C**). Interestingly, for the chAb 18B7-P1 and 18B7dg-P1 complexes, most of the contributions to activation energy of the transition state ΔΔG^≠^ (**Figure 5A**) are from the encounter step ΔΔG1^≠^ (**Figure 5B**). Hence, deglycosylation leads to differences in the energies of binding with respect to the glycosylated form. These differences are more accentuated for the encounter activation energy of chAb 18B7, indicating that the transition state for this complex is energetically more favorable than for the mAb 18B7dg-P1 complex at temperatures ranging from 20°C to 35°C (293 K to 308 K). In summary, kinetic and thermodynamic calculations indicate that deglycosylation does not affect the affinity of binding, but a heterologous associated C region will, as shown above.

### Molecular modeling analysis

**Figure 6:**
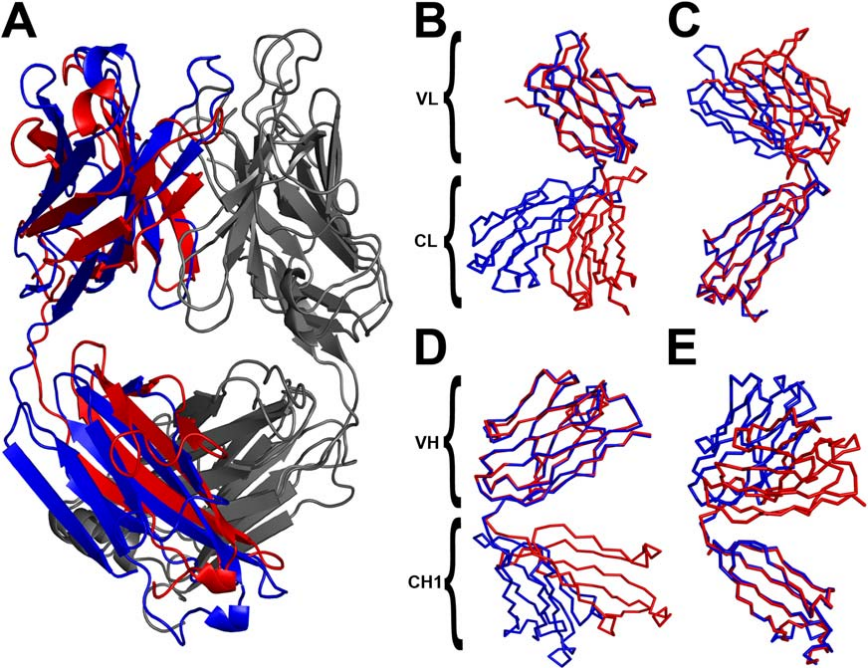
Structural models for chAb18B7 (red) and murine mAb18B7 (blue). *A*, Global structural superposition of the models; light chain depicted in gray. *B*, Structural superposition of light chain keeping fixed V_L_ and *C*, C_L_ domain. Structural superposition of *D*, heavy chain keeping fixed V_H_ and *E*, C_H1_ domain. Figures 6 and 7 were generated using PyMOL (http://pymol.sourceforge.net).

At a first glance, the backbone conformation of mAb 18B7 and chAb 18B7 models differed significantly (**Figure 6A**). However, a detailed analysis showed that these changes were due to different Ab elbow angles, i.e. angle between variable (V_L_ and V_H_) and C domains (C_L_ and C_H1_). If any of the two domains, V or C, for any of the chains, H and L, is kept fixed during structural superposition, a good structural agreement can be observed (L chain: **Figure 6B and 6C** keeping V_L_ and C_L_ fixed during structural superposition; H chain: **Figure 6D and 6E** superposing domain V_H_ and C_H1_ respectively).

**Figure 7:**
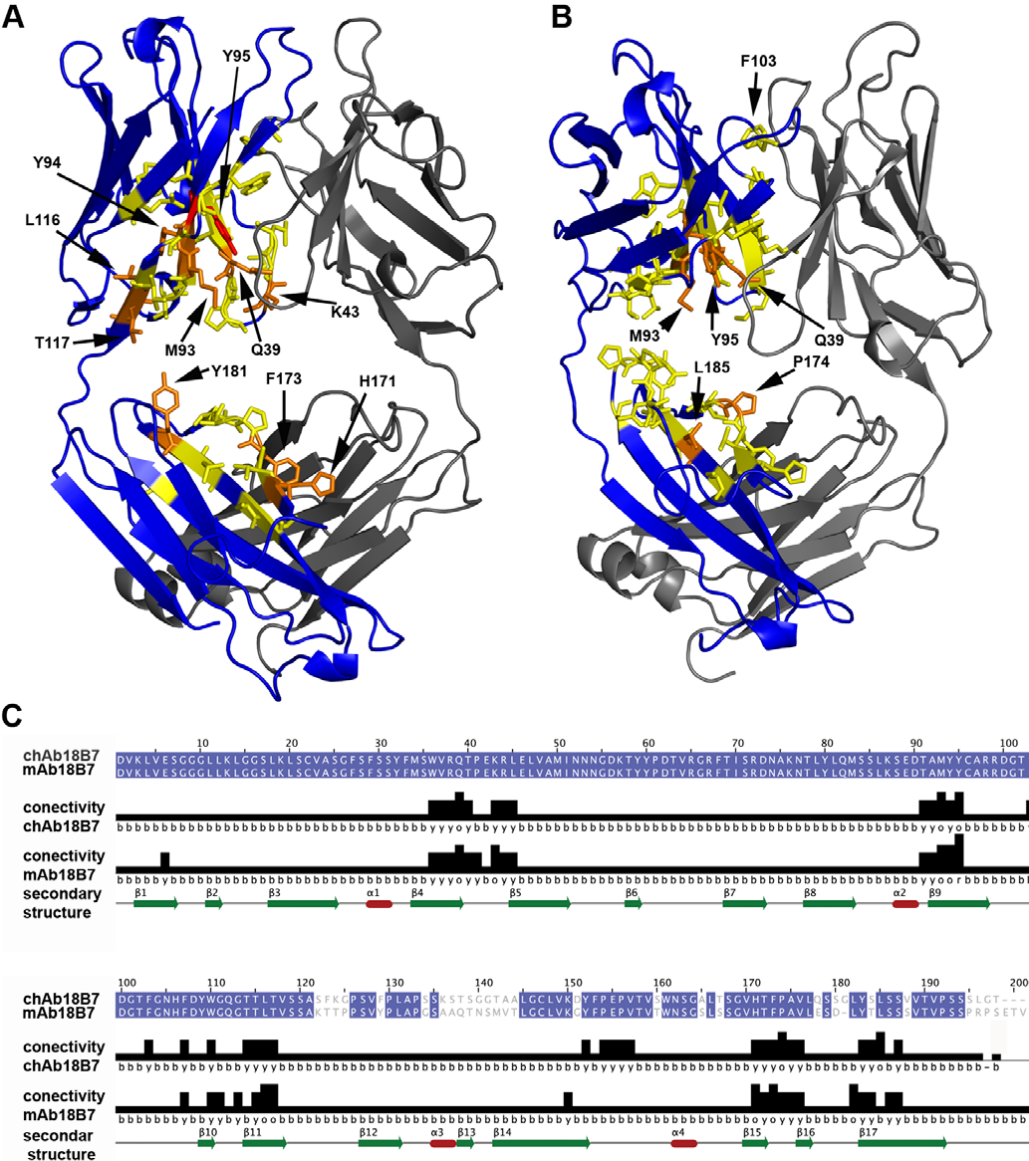
Connectivity analysis of residues in mAb 18B7 and ch18B7 models of C_H1_ domain. Cartoon representation of *A*, murine mAb 18B7 V_H_-C_H1_ and *B*, chAb 18B7. Areas of high connectivity are indicted by red and orange (red Zscore>2.0; orange: 1.5≤Zscore<2.0); medium connectivity residues are indicated in yellow (1.0≤Zscores<1.5), whereas low connectivity is indicated in blue (Zscore<1.0). Light chain is shown in gray. Side chains of amino acids with high connectivity values (Zscore≥1.5) are indicated by arrow. *C*, Residues masked in blue in the sequence alignment of H chain are equal in both Abs (domain V_H_: 1–120; domain C_H1_: 121–200). Connectivity values for each residues are shown in the alignment (in form of histogram and color coding, being b, y, o, and r: blue, yellow, orange, and red respectively); secondary structure elements are also shown.

Analysis of the connectivity of the Fab for murine mAb 18B7 and chAb 18B7 showed that these differences were located mainly at the interface between the C_H_ and V_H_ domains (**Figure 7A and 7B**). Highly connected residues (i.e. Zscore≥1.5) were assembled forming a network that spans from the C_H1_ domain to the V_H_ domain. This network can provide a logical explanation for a long-range allosteric effect that involves residue-to-residue communication. It is worth noting that Phe^103^ (**Figure 7B**) is located in one of the CDRs loop and the network of highly connected residues extends to the C_H1_ domain. **Figure 7C** shows the sequence alignment and the connectivity analysis; chAb 18B7 and mAb 18B7 display a differential connectivity pattern. This difference in connectivity pattern could explain the differential affinity of mAb 18B7 and chAb 18B7 toward an Ag. The analysis also suggests that these conformational variations are a consequence of species class-specific constraints.

### Polyreactive patterns of Abs 18B7

**Figure 8:**
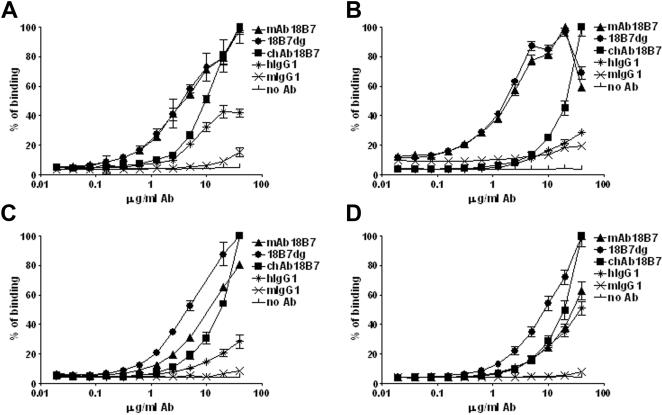
Reactivity patterns of mAb 18B7, 18B7dg and chAb 18B7 against polyreactive antigens. Panels correspond to the binding profiles of these Abs to, *A*, tubulin, *B*, ssDNA, *C*, thyroglobulin and *D*, actin. Human (hIgG1) and mouse (mIgG1) IgG1 were used as control. Data represent mean±SD of three measurements.

The polyreactive properties of mAb 18B7, 18B7dg and chAb 18B7 were measured by a panel of self-antigens consisting of actin, tubulin, thyroglobulin, and single-stranded DNA (**Figure 8A–D**). Polyreactivity was evident among these mAbs, showing differences in binding reactivity within the Ags used for the study. It is noticeable that chAb 18B7 has an overall lower binding reactivity for the self-Ags used in this study, in accordance with differences in specificity for this Ab with respect to mAb 18B7. In addition, we have shown that cross-reactivity does not only occur with closely related molecules because the studied cross-reactants are not related to the capsular polysaccharide GXM.

## Discussion

In previous studies, we established that a family of V-region identical murine IgG1, IgG2a, IgG2b, and IgG3 manifested diverse binding affinity and specificity that were attributed to C region effects on the V region. This effect was attributed to amino acid changes in C_H1_ that caused structural differences in the attached V region that translated into variations in antigen binding [11]. An implication of those results was that chAbs might also manifest differences in Ag binding as a consequence of amino acid sequence dissimilarities between heterologous C regions. Consistent with this notion a prior study comparing the binding of mAb 18B7 derived chAbs revealed differences in Ag binding but the results were only strongly suggestive, since avidity contributions resulting from Ab geometry and flexibility could have influenced the types of Ab-Ag complexes formed [1]. To rigorously establish whether heterologous C regions affected V region interactions with Ag it was necessary to use an experimental system whereby each V region interacted with only one epitope to avoid avidity contributions combined with a sensitive measure for detecting binding differences. To investigate the characteristics of binding we used SPR, a method that can detect the interaction of mAb immobilized on a sensor chip with a small antigen such as a peptide, thus avoiding avidity contributions and allow examination of the kinetics and thermodynamics of Ab-Ag complex formation.

A characteristic of all IgGs is glycosylation in the C_H2_ domain at Asn^297^ [12], which is necessary for structure recognition and stability [13], and several effector functions, such as complement binding and activation, and interactions with Fc receptors [14]–[18]. Glycosylation can affect the conformation of some Ab molecules by increasing or decreasing the stability of amino acid residues in the Ag binding site [19], [20]. Furthermore, numerous studies have shown that C_H2_ domain associated oligosaccharides play an essential role in IgG1-mediated Ab effector function [14], [15], [21]–[23]. However, few studies have addressed the issue of how differences in the structure of the C_H_ domains influence Ab-Ag binding [15].

Given the differences observed in binding affinities for the Fab molecules observed in our previous work [11], we could not rule out contributions of the C_H2_ domain to the Ab-Ag interaction. We have approached this issue by removing glycan chains using endoglycosidase digestion with a method known to be very effective in deglycosylating this mAb [24]. mAb 18B7 has a conventional IgG glycosylation pattern with a single *N*-linked glycosylation at Asn^263^ residue [24]. Circular dichroism spectral analysis of parental mAb 18B7 and 18B7dg revealed no major change in the secondary structure as a result of *N*-glycan removal, and their binding patterns to *C. neoformans* are essentially identical [24]. Native and deglycosylated mAb 18B7 binding to peptide P1 manifested similar binding kinetics and thermodynamics, suggesting that structural changes imparted upon C_H2_ domain by glycosylation do not extend into the V region and had little or no effect on the binding site of the murine IgG1 used for this study. This result is consistent with, and complementary of, the findings observed with anti-GXM mAbs 3E5, in that most of the isotype-related contributions to V-region binding affinity are a result of conformational changes on the C_H1_ domain, rather than C_H2_ [11].

In contrast to the similar interactions measured for the native and deglycosylated murine mAb 18B7, we found significant kinetic and thermodynamic differences in the comparison between mAb 18B7 and chAb 18B7. The V region of mAb 18B7 manifested a higher binding affinity when attached to a human γ1 chain. chAb 18B7 had a greater affinity, as determined by the equilibrium association constant than the parental murine mAb and different kinetics of binding, suggestive of differences in their fine specificity. These results imply that the nature of the C region electrostatic and hydrophobic interactions with the attached V region modulates the affinity and specificity of binding to Ag among Abs with an identical variable region. Consistent with these findings, others have reported that the immunogenicity of an Ab can be influenced by regions adjacent to the V domains [25], [26].

The stability of Ab-Ag complexes is regulated by the same forces as in other biological structures, namely electrostatic interactions and the hydrophobic effect [27], [28]. The structural modeling revealed that m18B7 and ch18B7 are structurally very similar, predicting only changes in the Ab elbow angles. Prior studies have speculated that the elbow angles might change in response of ligand binding [29], but no convincing experimental data has been found to support that hypothesis [30]. We favor the view that conformational differences imposed by the C_H1_ domain on the Ag-binding site could affect binding affinity and specificity. These conformational differences are probably a result of small changes in the electrostatic interactions between amino acid residues rather than by ‘gross’ conformational changes caused by hinge flexibility or by structural constrains imposed by the oligosaccharide moieties [31], [32].

Identifying functional residues is a complex issue. Activity can be modulated by residues that are distant from the binding site and these interactions within and between functional sites are crucial for protein activity. The study of closeness values in protein structure has proved useful in characterizing functional sites [33]. Such studies are also useful for describing the interaction networks within protein structures that are connected to active site residues, as those found in enzymes where effectors bind to residues distant from the active sites. Central residues have high closeness values and they are assumed to transmit and efficiently integrate in formation to the rest of the protein [33]. Our earlier studies [11] suggested that highly connected residues may be involved in the differences in fine specificity in four murine mAbs expressing different C_H_ regions and identical V regions. As observed in the homology models, an electrostatic network between the tyrosines residues interacting at the interface of the V region and the C_H1_ domain of murine mAb 18B7 could be more unfavorable for the net electrostatic forces of binding. These interaction networks formed at the C_H1_ domain appear to be directly responsible for the differences in affinity and specificity between these mAbs.

The differences observed in the specificity of these mAbs also affected the characteristics of binding to a panel of self-antigens, indicating that polyreactivity is not only preserved after Ig class switch [34], but it also depends on the contribution of structural changes caused by the C_H_ domains. This finding implies that polyreactivity is influenced by the surrounding C_H_ structures, possibly making the Ab-combining site more plastic, thus allowing these Abs to recognize a variety of Ags. Consequently, the structural heterogeneity conferred by the different C_H_ regions may result in the production of polyreactive Ab by changing charge and/or hydrophobicity of the V region. This implies that mouse-human chAb construction may yield Igs with binding characteristics that are different from the parental murine mAb, including the possibility for unexpected self-reactivity.

In summary C_H_ region glycosylation does not affect Ag binding, but exchanging murine and human C_H_ domains can have profound effects on affinity and specificity. Although the generalizability of the observations with mAb 18b7 and chAb 18B7 to other chimeric Abs is unknown, our findings suggest caution with assuming that simply replacing the C region domain maintains the specificity and affinity of the V region. Given that different V region genes encode for proteins with differences in protein sequence, Abs encoded by different V region genes may vary in their susceptibility to C region structural effects. Furthermore, we anticipate that these effects might also be expected to occur in chAbs composed of human V and mouse C regions [35]. Substituting the mouse C regions with human C regions in chAbs was a major advance in reducing the immunogenicity of mouse mAbs but our results show that this approach can have unpredictable effects on the thermodynamics of Ag-Ab complexes and the self-reactivity of chAbs. We do not know if the new reactivities exhibited by chAb 18B7 relative to the parent mAb 18B7 are clinically relevant, but the fact that polyreactivity occurs is the need for additional caution when examining the potential for cross-reaction with host tissues. Since most of the Abs currently used in drug therapy are of the IgG1 isotype it is important to understand how substituting a γ1 C_H_ chain affects murine V region binding affinity and specificity. An improved understanding of the mechanisms underlying this effect may allow the design of less immunogenic Abs for therapeutic use that maintain greater Ag-binding fidelity and lower polyreactivity.

## Methods

### MAbs and peptides

GXM-binding mAb 18B7 was made from a mouse immunized with a GXM-Tetanus Toxoid conjugate vaccine [8]. Mouse-human chAb 18B7 (IgG1) was generated by cloning and transfection as described [36]. mAb 18B7 was purified by protein G affinity chromatography (Pierce) from hybridoma culture supernatants, and dialyzed against at least two changes of PBS. Mouse-human chAb 18B7 was purified by protein L agarose beads (Sigma). mAb 18B7 was deglycosylated as described [24]. Briefly, 337 µg of mAb 18B7 were incubated at 37°C for 24 h with 100 mU of PNGaseF (Glyco). Then, the digestion mixture was dialyzed against at least two PBS changes. All Abs were analyzed by polyacrylamide gel electrophoresis to verify their integrity and correct molecular weight. Ab concentration was determined by ELISA and Bradford measurements. Peptide mimetic of GXM P1 (SPNQHTPPWMLK) [3] was synthesized and biotinylated by the Laboratory for Macromolecular Analysis at the Albert Einstein College of Medicine.

### Immobilization of mAb 18B7, 18B7dg and chAb 18B7

The BIAcore 3000 system and the research grade sensor chip CM5 (BIAcore) were used. mAbs were immobilized in the surface of the CM5 chip through primary amino groups using reactive esters. First, the carboxylated matrix was activated with 70 µl of a 1∶1 mixture of N-ethyl-N′(dimethylaminoporpyl)-carbodiimide (EDC) (Pierce) and N-hydroxysuccinimide (NHS) (Pierce). Then, mAbs 18B7 and 18B7dg were injected at 25 µg/ml in 10 mM 2-(N-morpholino) ethanesulphonic acid (MES) (Sigma-Aldrich), pH 6.0. chAb 18B7 was injected at 25 µg/ml in 10 mM sodium acetate (Sigma-Aldrich), pH 5.42. After mAb injection, remaining NHS-ester groups were blocked by injection of 70 µl of 1M ethanolamine (Sigma-Aldrich), pH 8.5.

### Kinetic Measurements

To analyze the kinetic and thermodynamic parameters of the Abs utilized in this study we used the peptide mimetic of GXM P1 [3]. The interaction of the mAbs-peptide complexes was analyzed at 10, 15, 20, 25, 30, and 35°C. Peptide P1 was tested at concentrations ranging from 1.56 µM to 200 µM at high flow rate (100 µl/min) in 0.2 mM K_2_HPO_4_/KH_2_PO_4_ pH 6.5, 150 mM KCl and 0.01% Tween 20. At the end of the dissociation phase the surface was regenerated by injecting 50 µl of 50 mM ethanolamine in 50% ethylene glycol (Sigma-Aldrich) and 50 µl of 10 mM HCl. An unmodified dextran surface was used as reference. Kinetic data was analyzed with the BIAevaluation 4.1 software. Data for each group was analyzed globally using a two-state model to obtain the forward (k_+1_, k_+2_) and reverse (k_−1_, k_−2_) rate constants. The rate constants for each global fit were used to calculate the association equilibrium constant, based on the following equations:
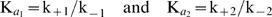
(1)

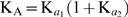
(2)The Gibbs free energy changes ΔG were calculated from the equilibrium constants:
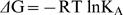
(3)

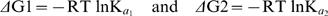
(4)

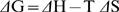
(5)These equations can be rearranged to yield the relation:
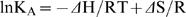
(6)


By measuring K_A_ as a function of temperature, one can plot ln K_D_ versus 1/T (eq. 6). Ideally, this plot, known as van't Hoff plot, should yield a straight line with a slope of −ΔH/R and an intercept ΔS/R. Enthalpy changes (ΔH) were calculated from the slope of the van't Hoff plot. Entropy changes (ΔS) were calculated from the equation 5, by substituting the values of ΔG (eq. 3) and assuming a constant ΔH with temperature. Equilibrium constants were calculated without considerations of the errors of the rate constants.

### Molecular modeling

mAb 18B7 and ch18B7 were modeled using the sequence for V_H_ and V_L_ from mAb 18B7 obtained from the protein GeneBank[37] with accession numbers AJ309266 and AJ309267, respectively. Each sequence was used to scan the Protein DataBank (PDB) [38] using PSI-BLAST [39] with default parameters. PSI-BLAST outputs were filtered using BlastProfiler [40] to select templates with the highest sequence coverage and sequence identity. Selected templates were manually inspected to choose those ones with the highest crystallographic quality and sequence coverage. The models were built with M4T [41] combining two experimental structures as templates for each sequence. A common structure for all sequences (PDB code 2h1p), was used as template for V and C regions of both L and H chain; and a specific template for the C_H1_ domain depending on the Ab species (PDB codes of 1sbs and 1pz5 C_H1_ domain, for human and mouse respectively). The 2h1p structure corresponds to the experimental three-dimensional structure of anti-GXM mAb 2H1 with peptide mimetic of GXM PA1. The average sequence identities between target sequences and templates were larger than 90%, assuring high quality models [42], [43]. The quality of the models was assessed using PROSA-II [44] and PROCHECK [45].

### Connectivity analysis of residues

Structural models of mAb 18B7 and chAb 18B7, were transformed into interaction graphs. The nodes of the graphs are the residues and the edges between nodes are any type of inter-atomic interactions, namely covalent (peptide bond) and non-covalent interactions (hydrogen bonds, polar interactions, and hydrophobic interactions). Inter-atomic interactions were described using the CSU program [46]. For each node (or residue) of the graph a *closeness centrality* Z-score was calculated as described in Amitai G. *et al* [33]. The *closeness centrality* is a measure of connectivity; a high closeness centrality value indicates a high number of interactions with the rest of nodes (or residues).

### ELISA

mAb 18B7, 18B7dg and chAb 18B7 were screened for their polyreactive properties against a panel of antigens by ELISA. Polystyrene plates were coated overnight at 4°C with actin (Sigma), tubulin (ICN) and thyroglobulin (Sigma) at 5 µg/ml in bicarbonate buffer pH 9.6, single stranded (ss) and DNA (Sigma) at 100 µg/ml in sodium citrate buffer, pH 6.0. Plates were blocked with 1% BSA in TBS for 2 h at 4°C. Abs were added at 40 µg/ml in 1% BSA/TBS and serially diluted. Plates were incubated overnight at 4°C. Ab binding was determined with alkaline-phosphatase conjugated goat anti-mouse H+L (Southern Biotech) for the murine Abs and alkaline-phosphatase conjugated goat anti-human IgG (Caltag) for chAb 18B7. The plates were developed with *p*-nitrophenyl phosphate substrate by measuring absorbance at 405 nm.

### Statistics

Standard deviations (SD) for the ELISA were calculated using Microsoft Excel functions for SD. The error bars on the van't Hoff plots correspond to the error propagation (ep) of the natural log (ep = SE_k_/k) of the standard error (SE) obtained with the BIAevaluation software for each rate constant individually.

## References

[1] McLean GR, Torres M, Elguezabal N, Nakouzi A, Casadevall A (2002). Isotype can affect the fine specificity of an antibody for a polysaccharide antigen.. J Immunol.

[2] Morelock M, Rothlein R, Bright S, Robinson M, Graham E (1994). Isotype choice for chimeric antibodies affects binding properties.. J Biol Chem.

[3] Torres M, May R, Scharff MD, Casadevall A (2005). Variable-region-identical antibodies differing in isotype demonstrate differences in fine specificity and idiotype.. J Immunol.

[4] McCloskey N, Turner MW, Steffner P, Owens R, Goldblatt D (1996). Human constant regions influence the antibody binding characteristics of mouse-human chimeric IgG subclasses.. Immunology.

[5] Schreiber JR, Cooper LJ, Diehn S, Dahlhauser PA, Tosi MF (1993). Variable region-identical monoclonal antibodies of different IgG subclass directed to Pseudomonas aeruginosa lipopolysaccharide O-specific side chain function differently.. J Infect Dis.

[6] Pollack M, Koles N, Preston M, Brown B, Pier G (1995). Functional properties of isotype-switched immunoglobulin M (IgM) and IgG monoclonal antibodies to Pseudomonas aeruginosa lipopolysaccharide.. Infect Immun.

[7] Larsen RA, Pappas PG, Perfect J, Aberg JA, Casadevall A (2005). Phase I evaluation of the safety and pharmacokinetics of murine-derived anticryptococcal antibody 18B7 in subjects with treated cryptococcal meningitis.. Antimicrob Agents Chemother.

[8] Casadevall A, Mukherjee J, Devi SJ, Schneerson R, Robbins JB (1992). Antibodies elicited by a Cryptococcus neoformans-tetanus toxoid conjugate vaccine have the same specificity as those elicited in infection.. J Infect Dis.

[9] Rothman RJ, Warren L, Vliegenthart JF, Hard KJ (1989). Clonal analysis of the glycosylation of immunoglobulin G secreted by murine hybridomas.. Biochemistry.

[10] Wright JF, Shulman MJ, Isenman DE, Painter RH (1990). C1 binding by mouse IgM. The effect of abnormal glycosylation at position 402 resulting from a serine to asparagine exchange at residue 406 of the mu-chain.. J Biol Chem.

[11] Torres M, Fernandez-Fuentes N, Fiser A, Casadevall A (2007). The immunoglobulin heavy chain constant region affects kinetic and thermodynamic parameters of antibody variable region interactions with antigen.. J Biol Chem.

[12] Sutton BJ, Phillips DC (1983). The three-dimensional structure of the carbohydrate within the Fc fragment of immunoglobulin G.. Biochem Soc Trans.

[13] Gala FA, Morrison SL (2002). The role of constant region carbohydrate in the assembly and secretion of human IgD and IgA1.. J Biol Chem.

[14] Tao MH, Morrison SL (1989). Studies of aglycosylated chimeric mouse-human IgG. Role of carbohydrate in the structure and effector functions mediated by the human IgG constant region.. J Immunol.

[15] Nose M, Wigzell H (1983). Biological significance of carbohydrate chains on monoclonal antibodies.. Proc Natl Acad Sci U S A.

[16] Wright A, Morrison SL (1994). Effect of altered CH2-associated carbohydrate structure on the functional properties and in vivo fate of chimeric mouse-human immunoglobulin G1.. J Exp Med.

[17] Radaev S, Sun PD (2001). Recognition of IgG by Fcgamma receptor. The role of Fc glycosylation and the binding of peptide inhibitors.. J Biol Chem.

[18] Butler M, Quelhas D, Critchley AJ, Carchon H, Hebestreit HF (2003). Detailed glycan analysis of serum glycoproteins of patients with congenital disorders of glycosylation indicates the specific defective glycan processing step and provides an insight into pathogenesis.. Glycobiology.

[19] Wallick S, Kabat E, Morrison S (1988). Glycosylation of a VH residue of a monoclonal antibody against alpha (1- ---6) dextran increases its affinity for antigen 10.1084/jem.168.3.1099.. J Exp Med.

[20] Wright A, Tao MH, Kabat EA, Morrison SL (1991). Antibody variable region glycosylation: position effects on antigen binding and carbohydrate structure.. Embo J.

[21] Wright A, Morrison SL (1997). Effect of glycosylation on antibody function: implications for genetic engineering.. Trends Biotechnol.

[22] Jefferis R, Lund J, Pound JD (1998). IgG-Fc-mediated effector functions: molecular definition of interaction sites for effector ligands and the role of glycosylation.. Immunol Rev.

[23] Hindley SA, Gao Y, Nash PH, Sautes C, Lund J (1993). The interaction of IgG with Fc gamma RII: involvement of the lower hinge binding site as probed by NMR.. Biochem Soc Trans.

[24] Wang F, Nakouzi A, Alvarez M, Zaragoza O, Angeletti RH (2006). Structural and functional characterization of glycosylation in an immunoglobulin G1 to Cryptococcus neoformans glucuronoxylomannan.. Mol Immunol.

[25] Knight DM, Wagner C, Jordan R, McAleer MF, DeRita R (1995). The immunogenicity of the 7E3 murine monoclonal Fab antibody fragment variable region is dramatically reduced in humans by substitution of human for murine constant regions.. Mol Immunol.

[26] Pritsch O, Hudry-Clergeon G, Buckle M, Petillot Y, Bouvet JP (1996). Can immunoglobulin C(H)1 constant region domain modulate antigen binding affinity of antibodies?. J Clin Invest.

[27] Lipschultz CA, Yee A, Mohan S, Li Y, Smith-Gill SJ (2002). Temperature differentially affects encounter and docking thermodynamics of antibody–antigen association.. J Mol Recognit.

[28] Braden BC, Poljak RJ (1995). Structural features of the reactions between antibodies and protein antigens.. Faseb J.

[29] Huber R, Deisenhofer J, Colman PM, Epp O, Fehlhammer H (1976). Proceedings: X-ray diffraction analysis of immunoglobulin structure.. Hoppe Seylers Z Physiol Chem.

[30] Stanfield RL, Zemla A, Wilson IA, Rupp B (2006). Antibody elbow angles are influenced by their light chain class.. J Mol Biol.

[31] Greenspan NS (2001). Dimensions of antigen recognition and levels of immunological specificity.. Adv Cancer Res.

[32] Greenspan NS, Rouvray DH (1997). Conceptions of epitopes and paratopes and the ontological dynamics of immunological recognition.. Concepts in Chemistry: A Contemporary Challenge.

[33] Amitai G, Shemesh A, Sitbon E, Shklar M, Netanely D (2004). Network analysis of protein structures identifies functional residues.. J Mol Biol.

[34] Fernandez C, Alarcon-Riquelme ME, Abedi-Valugerdi M, Sverremark E, Cortes V (1997). Polyreactive binding of antibodies generated by polyclonal B cell activation. I. Polyreactivity could be caused by differential glycosylation of immunoglobulins.. Scand J Immunol.

[35] Valenzuela DM, Murphy AJ, Frendewey D, Gale NW, Economides AN (2003). High-throughput engineering of the mouse genome coupled with high-resolution expression analysis.. Nat Biotechnol.

[36] McLean GR, Nakouzi A, Casadevall A, Green NS (2000). Human and murine immunoglobulin expression vector cassettes.. Mol Immunol.

[37] Benson DA, Karsch-Mizrachi I, Lipman DJ, Ostell J, Wheeler DL (2006). GenBank.. Nucleic Acids Res.

[38] Berman HM, Westbrook J, Feng Z, Gilliland G, Bhat TN (2000). The Protein Data Bank.. Nucleic Acids Res.

[39] Altschul SF, Madden TL, Schaffer AA, Zhang J, Zhang Z (1997). Gapped BLAST and PSI-BLAST: a new generation of protein database search programs.. Nucleic Acids Res.

[40] Rai BK, Fiser A (2006). Multiple mapping method: a novel approach to the sequence-to-structure alignment problem in comparative protein structure modeling.. Proteins.

[41] Fernandez-Fuentes N, Madrid-Aliste CJ, Rai BK, Fajardo JE, Fiser A (2007). M4T: a comparative protein structure modeling server.. Nucleic Acids Research.

[42] Fiser A, Sali A (2003). Modeller: generation and refinement of homology-based protein structure models.. Methods Enzymol.

[43] Baker D, Sali A (2001). Protein structure prediction and structural genomics.. Science.

[44] Sippl MJ (1993). Recognition of errors in three-dimensional structures of proteins.. Proteins.

[45] Laskowski RA, MacArthur MW, Moss DS, Thornton JM (1993). PROCHECK: a program to check the stereochemical quality of protein structures.. J Appl Cryst.

[46] Sobolev V, Sorokine A, Prilusky J, Abola EE, Edelman M (1999). Automated analysis of interatomic contacts in proteins.. Bioinformatics.

